# Mediation effects of thyroid function in the associations between phthalate exposure and lipid metabolism in adults

**DOI:** 10.1186/s12940-022-00873-9

**Published:** 2022-07-01

**Authors:** Han-Bin Huang, Po-Keng Cheng, Chi-Ying Siao, Yuan-Ting C. Lo, Wei-Chun Chou, Po-Chin Huang

**Affiliations:** 1 School of Public Health, National Defense Medical Center, Taipei, Taiwan; 2 National Institute of Environmental Health Sciences, National Health Research Institutes, Miaoli County, Taiwan; 3 Department of Environmental and Global Health, University of Florida, Gainesville, USA; 4 Research Center for Environmental Medicine, Kaohsiung Medical University, Kaohsiung, Taiwan; 5 Department of Medical Research, China Medical University Hospital, China Medical University, Taichung, Taiwan

**Keywords:** Phthalates, Metabolic effect, Thyroid function, Mediating effect, Lipid metabolism

## Abstract

Phthalates are a group of industrial chemicals widely used in everyday products including cosmetics, food packaging and containers, plastics, and building materials. Previous studies have indicated that urinary phthalate metabolites are associated with metabolic effects including those on lipid metabolism, but the results are mixed. Furthermore, whether thyroid function mediates the association between phthalate exposure and lipid metabolism remains unclear. In the present study, we explored whether changes in thyroid function markers mediate the associations between phthalate exposure and lipid metabolism indicators in Taiwanese adults. The cross-sectional data were obtained from the Taiwan Environmental Survey for Toxicants conducted in 2013. Levels of 11 urinary phthalate metabolites, levels of 5 thyroid hormones, and 8 indicators of lipid metabolism were assessed in 222 Taiwanese adults. The relationships of urinary phthalate metabolite levels with serum thyroid hormone levels and lipid metabolism indicators were explored using multiple regression models. Mediation analysis was conducted to evaluate the role of thyroid function in the association between phthalate exposure and lipid metabolism. The metabolite of di(− 2-ethylhexyl) phthalate (∑DEHPm) exhibited a significant positive association with the lipid metabolite indicator of high-density lipoprotein cholesterol (HDL-C; β = 0.059, 95% confidence interval [CI] = 0.009, 0.109) in adults, and the thyroid function indicator thyroxine (T_4_) had a significant negative association with the metabolite ∑DEHPm (β = − 0.059, 95% CI = − 0.101, − 0.016) and a significant negative association with HDL-C (β = − 0.284, 95% CI = − 0.440, − 0.128). The T_4_ indirect effect was 0.015 (95% CI = − 0.0087, 0.05), and the mediation effect was 32.2%. Our results support the assumption that exposure to phthalates influences the homeostasis of lipid metabolism by interfering with thyroid function.

**Supplementary Information:**

The online version contains supplementary material available at 10.1186/s12940-022-00873-9.

## Introduction

Plastics have become an essential part of everyday life. Plasticizers are indispensable substances in the manufacture of plastic goods. They are used as lubricants, stabilizers, and flavoring agents in various products, including medical devices, pharmaceuticals, personal hygiene products, food packaging, and containers. In Taiwan, a plasticizer scandal occurred on 2011 when the Taiwan Food and Drug Administration detected plasticizers in a batch of probiotic ingredients, which led to revelations that unscrupulous manufacturers were including inedible additives (instead of clouding agents) in beverages, pastries, bread, medicines, and other consumables to save costs [1]. This caused shock and panic throughout Taiwan. Although the scandal receives less attention today, plasticized products that are hazardous to health are still around us and threaten the next generation.

Plasticizers are wide ranging, with the commonly used type of compound being phthalates such as di-(2-ethylhexyl) phthalate (DEHP), butyl benzyl phthalate (BBzP), diethyl phthalate (DEP), dibutyl phthalate (DBP), dimethyl phthalate (DMP), di-isononyl phthalate (DINP), di-n-octyl phthalate (DNOP), di-isodecyl phthalate (DIDP). In Taiwan, these common types of phthalates are regulated by the Environmental Protection Administration’s Poisons and Chemicals Bureau and are classified as toxic chemical substances (categories 1 and 2) with the characteristics of being difficult to decompose and chronically toxic. Among these common types of phthalates, DEHP was the most widely used phthalate in Taiwan at 2013, and it is often added to food containers, construction materials, medical devices, and toys. DEHP is colorless and odorless at room temperature and pressure and is a water-insoluble, fat-soluble, viscous liquid with a molecular weight of 390.56. Its molecular formula is C_6_H_4_(C_8_H_17_COO)_2_. The International Agency for Research on Cancer classified DEHP as a 2B human carcinogen, which led the European Union (EU) to restrict its use in toys in 1999 [2].

Phthalates, also known as endocrine disruptors, may have a profound effect on body metabolism and exert related metabolic effects. Bajkin et al. suggested that prolonged low-dose phthalate exposure poses a health risk either through interference with endocrine system effects (anti-androgen, thyroxine) or gene expression, leading to systemic diseases such as abdominal obesity [3]. In an animal study, phthalates were discovered to impair reproductive function or cause liver tumors [4]. Exposure to phthalates like DEHP can affect thyroid signaling by interfering with thyroid-stimulating hormone (TSH) receptors, binding to transporter proteins, and altering thyroid follicle cells’ iodine uptake through several potential mechanisms [5, 6]. Experimental data from animal cells indicate that exposure to phthalates alters adipogenesis and lipid metabolism [7]. Exposure to phthalates also promotes weight gain by binding to the peroxisome proliferator–activated receptor (PPAR), which regulates fatty acid storage [8].

Thyroid function indicators are TSH, triiodothyronine (T_3_), thyroxine (T_4_), free triiodothyronine (free T_3_), and free thyroxine (free T_4_). These hormones regulate the body’s energy metabolism, growth, development, and reproductive system. In a study of phthalate exposure and indicators of thyroid function in adults, negative correlations were observed between urinary DEHP metabolites and T_3_ as well as free T_4_ [9]. Another study noted a negative correlation between DEHP and T_4_ in adults but a positive association between T_4_ and DEHP in adolescents [10]. Similar results have been reported in other studies. For example, Huang et al. discovered that DEHP metabolites were negatively correlated with free T_4_ and T_4_ in adults, but BBzP metabolites were positively assocaited with free T_4_ in minors [11]. A study in children revealed a positive relationship between free T_3_ and DEHP metabolites and negative relationships of both DBP and BBzP metabolites with T_4_ [12]. A Korean study demonstrated that DEHP metabolites were only negatively correlated with T_4_ in men; in women, DBP and BBzP metabolites were each negatively correlated with both TSH and T_3_ [13]. Phthalate exposure is also correlated with thyroid function indicators in pregnant women and children [14]. However, another study noted no such association with these indicators [15].

In addition, high-density lipoprotein cholesterol (HDL-C), low-density lipoprotein cholesterol (LDL-C), triglyceride (TG), and total cholesterol (TC) are indicators of lipid metabolism. The Castelli risk index I (CRI-I), Castelli risk index II (CRI-II), non-HDL cholesterol (NHC), and atherogenic coefficient (AC) are indexes derived from the lipid ratio. These indicators (and thyroid function indicators) involve potentially complex physiological mechanisms. Thyroid hormones play a key role in regulating body metabolism; for example, thyroid hormones stimulate lipid synthesis and promote lipolysis [16]. The aforementioned biological indicators are also related to obesity, cerebrovascular disease, and cardiovascular disease. In recent years, the effects of abnormal lipid metabolism and physiological effects have attracted considerable research attention. Tóth et al. using the National Health and Nutrition Examination Survey 2003–2006 reported an estimated 53% of U.S. adults have lipid abnormalities: 27% have high LDL-C, 23% have low HDL-C, and 30% have high TG, in which 21% of U.S. adults have mixed dyslipidemia (high LDL-C with either low HDL-C and/or high TG), with nearly 6% having all three lipid abnormalities [17]. High LDL-C, high TC, high TG, and low HDL-C levels along with hypertension and obesity are considered risk factors for stroke and cardiovascular disease [18–20]. Epidemiological studies demonstrated a correlation between indicators of thyroid function and biological indicators of lipid metabolism, and phthalates may interfere with thyroxine and affect the regulation of metabolism [3]. This suggests that changes in thyroid function may cause imbalances in body regulation of metabolism, and that these biological indicators are pivotal factors in the prevention of disease. Our previous study indicated exposure to phthalates may affect thyroid function to increase the risk of insulin resistance, and free T_4_ acted as a mediating factor that affects insulin resistance [21]. Thus, exploring early biomarkers involved in exposure to phthalates and lipid metabolism is warrant. Therefore, elucidating the changes in thyroid function and lipid metabolism indicators caused by phthalates is critical.

## Materials and methods

### Study participants

This cross-sectional study investigated subsamples of participants to the Taiwan Environmental Survey for Toxicants (TEST) [21–23]. We cooperated with Taiwan National and Nutrition Health Survey team (NAHSIT) to employ the same sampling method and participant recruitment strategy. For NAHSIT, participants from all age groups and 20 major cities or counties in Taiwan were selected. Each Taiwanese city or township is divided into two groups according to urbanization and population density. A city or county is represented by two townships selected at random from each group. Individuals who had a severe illness (e.g., cancer), were pregnant or breastfeeding, were imprisoned or hospitalized, or were not Taiwanese nationals were excluded from the study. All of the participants were at least 7 years old and from Taiwan. The TEST results from May through December 2013 for 11 Taiwanese cities or counties were included. We interviewed a total of 500 subjects on the day of health examination at community center or elementary school; 394 subjects participated in this study, which yielded a response rate around 78%.

**Fig. 1:**
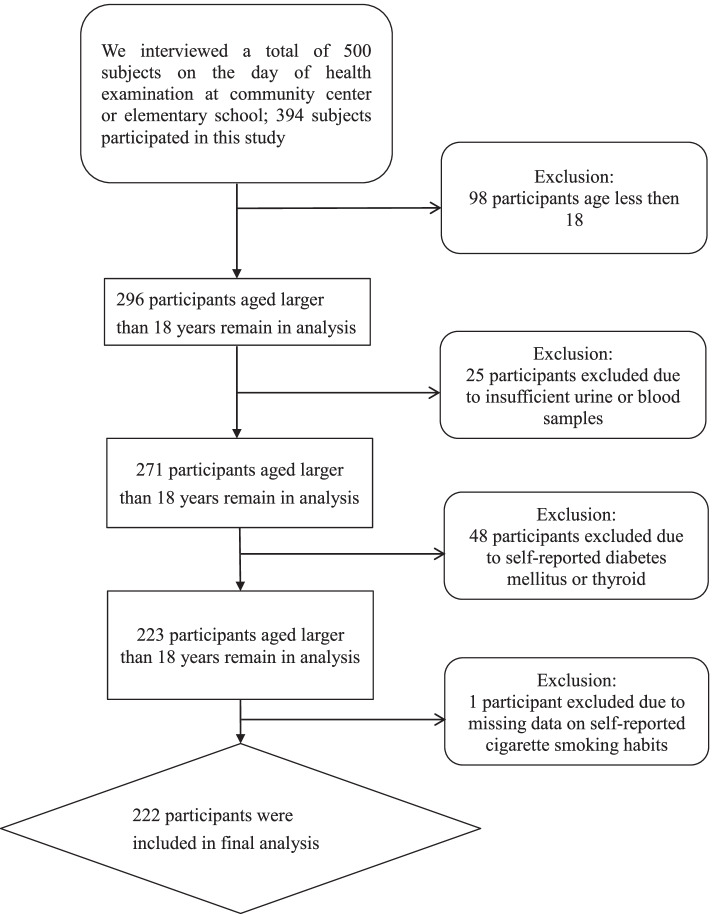
Flow chart of the recruitment of the study

Before enrolment, all individuals provided informed consent and had been volunteers who had joined NAHSIT. This study comprised 296 TEST participants aged larger than 18 years, with 25 participants excluded due to insufficient urine or blood samples, 48 participants excluded due to self-reported diabetes mellitus or thyroid dysfunction, and 1 participant excluded due to missing data on self-reported cigarette smoking habits. A total of 222 participants were included in the final analysis (Fig. 1). Individual characteristics (e.g., sex, age, and BMI) as well as lifestyle exposures (e.g., alcohol and cigarette usage) were collected via a questionnaire. This study was approved by the National Health Research Institutes’ Research Ethics Committee at Taiwan (no. EC1020206).

### Measurement of urinary phthalate metabolite level

Participant’s morning spot urine was analyzed for seven phthalates, namely DEHP, DiBP, DnBP, DEP, DMP, DINP, and BBzP. Additionally, 11 urinary phthalate metabolites were assessed: mono-(2-ethyl-5-hydroxyhexyl) phthalate (MEHHP), monoethylhexyl phthalate (MEHP), mono-(2-ethyl-5-carboxypentyl) phthalate (MECPP), mono-(2-ethyl-5-oxo-hexyl) phthalate (MEOHP), monoethyl phthalate (MEP), mono-n-butyl phthalate (MnBP), mono-(2-carboxymethylhexyl) phthalate (MCMHP), monoisobutyl phthalate (MiBP), monobenzyl phthalate (MBzP), monomethyl phthalate (MMP), and monoisononyl phthalate (MiNP). Phthalate metabolites concentrations were measured using online liquid chromatography instrument and an Agilent 1200/API 4000 tandem mass spectrometry system (Applied Biosystems, Foster City, CA, USA) [22, 24], detailed information is included in Supplementary materials. Furthermore, the sums of the DEHP metabolite (ΣDEHPm) and DBP metabolite (ΣDBPm) molar concentrations were derived [25, 26]. For MEOHP, MECPP, MBzP, MEP, MMP, and MEHHP, the limit of detection (LOD) was 0.3 ng/mL. Furthermore, the LOD for MiBP and MnBP was 1.0 ng/mL, the LOD for MiNP and MCMHP was 0.1 ng/mL, and the LOD for MEHP was 0.7 ng/mL. When the phthalate metabolite levels were less than the LOD, we used half of the LOD value as a substitute [27]. Each batch of analyzed samples contained a blank, repeated quality control (QC) sample. The QC sample for each sample batch in pooled urine samples was spiked with a mixture of phthalate metabolite standards (20–50 ng/mL). The QC sample’s relative percentage difference was to be less than ±30%, and QC sample’s recovery rate was to be 100% ± 20% [28].

### Levels of thyroid hormones and lipid metabolism indicators

Thyroid function (e.g., T_3_, T_4_, free T_4_, thyroxine-binding globulin [TBG], and TSH levels) and lipid metabolism indicators (e.g. HDL-C, LDL-C, TG, and TC levels) were assessed in all participants by using a morning fasting blood test. A chemistry analyzer (Beckman Coulter Unicel DxC 800), chemiluminescent microparticle immunoassay system (Beckman Coulter Inc., Brea, CA, USA), and immunoenzymometric assay (Monobind Inc., Product Code 3525–300) were employed to assess serum thyroid hormone (i.e., T_3_, T_4_, free T_4_, and TSH), TBG, HDL-C, LDL-C, TG, and TC levels (detailed information is included in Supplementary materials). The aforementioned analyses of thyroid function were randomly performed in Taiwan Accreditation Foundation-certified laboratories (no. 1447 and 1673), which are recognized by the International Laboratory Accreditation Cooperation Mutual Recognition Arrangement, by a technician blinded to the participants’ thyroid condition [11, 29]. The majority of thyroid hormone levels in our population were inside the reference ranges. The adults with T_3_, T_4_, free T_4,_ TSH, and TBG levels that were outside the reference ranges accounted for 2.7, 4.5, 6.8, 5.0, and 29.3% of the participants, respectively. In addition, the adults with T_3_, T_4_, free T_4,_ TSH, and TBG levels that were below the reference ranges accounted for 0.9, 3.6, 6.3, 0.9, and 10.8% of the participants, whereas the adults with T_3_, T_4_, free T_4,_ TSH, and TBG levels that were above the reference ranges accounted for 1.8, 0.9, 0.5, 4.1, and 18.5% of the participants. Due to the lack of information, the reference range for TBG was for Caucasian which may result in the high percentage exceeding rate here. The adults with TC, HDL-C, LDL-C, and TG levels outside the reference range constituted 39.2, 9.0, 29.7, and 22.1% of the participants, respectively. The CRI-I, CRI-II, AC, and NHC were calculated using the following formulas. CRI-I was derived by dividing TC by HDL-C, and CRI-II was derived by dividing LDL-C by HDL-C [30]. NHC was derived by subtracting TC by HDL-C [31], and AC was derived by subtracting TC by HDL-C and then divided by HDL-C [32].

1 $$\mathrm{CRI}-\mathrm{I}=\mathrm{TC}\div \mathrm{HDL}-\mathrm{C}$$

2 $$\mathrm{CRI}-\mathrm{II}=\mathrm{LDL}-\mathrm{C}\div \mathrm{HDL}-\mathrm{C}$$

3 $$\mathrm{NHC}=\mathrm{TC}-\mathrm{HDL}-\mathrm{C}$$

4 $$\mathrm{AC}=\left(\mathrm{TC}-\mathrm{HDL}-\mathrm{C}\right)\div \mathrm{HDL}-\mathrm{C}$$

CRI-I, also known as the cardiac risk ratio, indicates the formation of coronary plaques and has a diagnostic value comparable to total cholesterol. CRI-II has been found to be an excellent predictor of cardiovascular risk [33]. CRI-I, CRI-II, and AC are ratios in predicting the risk of coronary artery disease [32], and NHC is a predictor for cardiovascular disease [34, 35].

### Statistical analysis

We first calculated the medians and GMs of the urinary phthalate metabolite levels, thyroid hormone levels, and lipid metabolism indicators. In multiple regression models, these urinary phthalate metabolite levels, lipid metabolism indicators, and thyroid hormone levels were transformed by natural logarithm to fulfill the normality assumption. Therefore, the estimated coefficients indicate the percent increase in the dependent variable for every 1% increase in the independent variable. We use sum molar concentrations of MEHP, MEHHP, MEOHP, MECPP, and MCMHP (ΣDEHPm) to represent for DEHP metabolites, which could prevent different results in models for individual DEHP metabolites. After adjusting for covariates, the associations between lipid metabolism indicators and phthalate exposure, thyroid hormones and phthalate exposure, and lipid metabolism indicators (dependent variables) and thyroid function (independent variables) were examined. The covariates (i.e., sex, age, BMI, and cigarette smoking) were selected based on relevant studies [10, 36] and a change in the estimated coefficient larger than 10% (i.e., TBG levels) [37]. TBG binds thyroid hormones in circulation, in which TBG could be potential confounder related to thyroid hormones homeostasis. To reduce intraindividual variation in spot urine tests, the level of urinary creatinine was also included as covariate [38]. The models applied for examining the association between phthalate exposure and lipid metabolism indicators were adjusted for BMI (continuous), sex (categorical), age (continuous), urinary creatinine levels (continuous), and cigarette smoking (categorical, have at least one cigarette smoking per day or not). The models applied for examining the association between phthalate exposure and thyroid hormones were adjusted for BMI (continuous), sex (categorical), age (continuous), TBG levels (continuous), urinary creatinine levels (continuous), and cigarette smoking (categorical). The models applied for examining the association between lipid metabolism indicators and thyroid function were adjusted for BMI (continuous), sex (categorical), age (continuous), TBG levels (continuous), and cigarette smoking (categorical). In addition, we also incorporate Bayesian kernel machine regression (BKMR) [39], a nonparametric approach for chemical mixtures, to explore the associations between lipid metabolism indicators and phthalate exposure, thyroid hormones and phthalate exposure, and lipid metabolism indicators and thyroid function. The R mediation package [40] was used to perform mediation analysis to evaluate indirect and direct effects and estimate the proportions of mediation when exposure of phthalate metabolite was significantly associated with distinct thyroid hormones or lipid metabolism indicators that had previously been significantly associated with each other (*p* = 0.05 as significance level in multiple linear regressions). Briefly, a mediation analysis is comprised of three sets of regression: X → Y, X → M, and X + M → Y. Suppose Y = b_01_ + b_1_X + e_1_, M = b_02_ + b_2_X + e_2_, Y = b_03_ + b_4_X + b_3_M + e_3_, the indirect effect is calculate as b_1_–b_4_ or b_2_ × b_3_ and direct effect is b_4_. The proportions of mediation is calculated as indirect effect divide by total effect b_1_. Details of mediation analysis have been described by Huang et al. [21]. R version 4.1.0 (R Foundation for Statistical Computing, Vienna, Austria) was used to conduct all statistical analyses.

## Results

### Demographic characteristics

In this study, 105 (47.3%) and 117 (52.7%) of the participants were male and female, respectively, with an average age of 52.2 years and an average BMI of 24.8. The majority of the participants were married (72.1%). Percentage of college graduates or senior high school was 60.8%. A total of 119 (55.9%) of the participants were from households with annual incomes of less than US$15,625, and 60 (28.2%) were from households with annual incomes between US$15,625 and US$31,250. In terms of daily personal habits, 52 (23.4%) of the participants smoked cigarettes, 28 (12.8%) consumed alcohol, 89 (40.1%) drank coffee, 134 (60.6%) drank tea, and 13 (5.9%) chewed betel nut. In terms of environmental exposure assessment and the daily use of plastic products, 50 of the participants (22.5%) had used pesticides at home in the past month, 68 (30.9%) lived within 1 km of farmland, and 145 (65.6%) and 165 (74.7%) had habits of eating fried and grilled foods, respectively. Among the participants, 26 (11.7%), 161 (72.5%), 124 (55.9%), and 167 (75.2%) usually used plastic tableware, plastic wrap, plastic containers, and plastic bags (for refrigeration or heating), respectively (Table 1).

**Table 1:** Demographic characteristics of the study population (*N* = 222)

Variables	Adults (≧18 years, N = 222)	
	Mean ± SD	
Age (years)	222	52.2 ± 17.5
BMI	222	24.8 ± 4.57
	N	%
Sex		
Female	117	52.7
Male	105	47.3
Region		
Northern Taiwan	64	28.8
Central Taiwan	34	15.3
Southern Taiwan	62	27.9
Eastern Taiwan	39	17.6
Remote islands	23	10.4
Marital status		
Single	42	18.9
Married	160	72.1
Divorce/widowed	20	9.0
Education		
Junior high school/elementary school (≤ 9 years)	87	39.2
Senior high school (9 ~ 12 years)	48	21.6
≧College/Graduates (≥ 12 years)	87	39.2
Household annual income (USD) ^a^		
< 15,625	119	55.9
15,625 ~ 31,250	60	28.2
≥ 31,250	34	15.9
Cigarette smoking ^b^		
Yes	52	23.4
Alcohol consumption ^c^		
Yes	28	12.8
Coffee drinking ^d^		
Yes	89	40.1
Tea drinking ^e^		
Yes	134	60.6
Betel nut chewing ^f^		
Yes	13	5.9
Pesticide use at home		
Yes	50	22.5
Lived near farmland (with 1 km) ^g^		
Yes	68	30.9
Habit of eating fried food ^h^		
Yes	145	65.6
Habit of eating grilled food ^i^		
Yes	165	74.7
Plastic tableware use		
Yes	26	11.7
Plastic wrap use for refrigeration or heating		
Yes	161	72.5
Plastic containers use for refrigeration or heating		
Yes	124	55.9
Plastic bags use for refrigeration or heating		
Yes	167	75.2

^a^ 9 missing values in household annual income; currency exchange rate of USD to new Taiwan dollar is 1:32

^b^ Participants smoking at least one cigarette per day

^c^ 4 missing values in alcohol consumption; participants consuming at least one bottle of alcohol per week

^d^ Participants consuming at least one cup of coffee per week

^e^ 1 missing values in tea drinking; participants consuming at least one cup of tea per week

^f^ Participants chewing at least one betel nut per week

^g^ 2 missing values in live near farm land

^h^ 1 missing value in habit of eating fried food

^i^ 1 missing value in habit of eating grilled food

### Concentrations of urinary phthalate metabolite, lipid metabolism indicators and thyroid hormones

Table 2 presents the concentrations of urinary phthalate metabolite levels, lipid metabolism indicators, and thyroid hormones. Most urine phthalate metabolites demonstrated high to moderate detection rates, with the exception of MiNP and MBzP, which showed low detection rates. Consequently, MiNP and MBzP were not included in final analysis. GMs of MiBP, MEP, MMP, MnBP, MEHP, MECPP, MEHHP, MCMHP, and MEOHP concentrations in urine were 3.60, 10.97, 23.05, 9.72, 3.90, 17.75, 15.84, 1.54, and 8.04 ng/mL, respectively. ΣDBPm and ΣDEHPm GMs were respectively 0.10 and 0.20 nmol/mL. The detection rates for all thyroid hormones and lipid metabolism indicators were 100%. GMs of thyroid hormones were 1.55 μIU/mL, 105.58 ng/dL, 7.26 μg/dL, 0.92 ng/dL, and 21.26 μg/mL for TSH, T_3_, T_4_, free T_4_, and TBG, respectively. GMs of lipid metabolism indicators were 106.84 mg/dL, 190.62 mg/dL, 57.30 mg/dL, 109.16 mg/dL, 3.33, 1.91, 129.88, and 2.27 for TG, TC, HDL-C, LDL-C, CRI-I, CRI-II, NHC, and AC, respectively. The adults with T_3_, T_4_, free T_4,_ TSH, and TBG levels that were inside the reference ranges accounted for 97.3, 95.5, 93.2, 95.0, and 70.7% of the participants, respectively. The adults with TC, HDL-C, LDL-C, TG, CRI-I, CRI-II, NHC, and AC levels inside the reference range constituted 60.8, 91.0, 70.3, 77.9, 82.9, 80.6, 48.6, and 77.0% of the participants, respectively.

**Table 2:** Distribution of urinary phthalate metabolites (ng/mL), thyroid hormone levels, and lipid metabolism indicators among adults

Variables	Adults (≧18 years) (N = 222)			
	N (%) > LOD	Inside reference range (%)	Median (P_25_-P_75_)	GM (95% CI)
Phthalate metabolites ^a^				
MMP	212 (95.5)	91.4	23.23 (10.34, 53.21)	23.05 (18.48, 28.76)
MEP	204 (91.9)	96.8	12.02 (5.33, 25.97)	10.97 (8.66, 13.88)
MiBP	158 (71.2)	96.4	7.07 (ND, 17.08)	3.60 (2.70, 4.79)
MnBP	193 (86.9)	97.3	14.76 (6.16, 28.27)	9.72 (7.53, 12.56)
MBzP	51 (23.0)	99.1	ND (ND, ND)	0.32 (0.27, 0.39)
MEHP	175 (78.8)	99.1	6.90 (3.11, 12.12)	3.90 (3.06, 4.96)
MEHHP	217 (97.7)	95.0	16.24 (9.82, 30.27)	15.84 (13.72, 18.28)
MEOHP	205 (92.3)	96.4	10.28 (5.58, 16.37)	8.04 (6.71, 9.62)
MECPP	214 (96.4)	95.9	20.17 (11.30, 32.60)	17.75 (15.06, 20.91)
MCMHP	142 (64.0)	98.2	3.21 (ND, 6.39)	1.54 (1.21, 1.96)
MiNP	23 (10.4)	99.5	ND (ND, ND)	0.21 (0.19, 0.24)
ΣDEHPm (nmole/mL) ^b^			0.20 (0.12, 0.33)	0.20 (0.18, 0.22)
ΣDBPm (nmole/mL) ^b^			0.11 (0.05, 0.20)	0.10 (0.08, 0.12)
Thyroid Hormones ^c^				
TSH (μIU/mL)	222 (100)	95.0	1.48 (1.09, 2.22)	1.55 (1.43, 1.69)
T_3_ (ng/dL)	222 (100)	97.3	108.00 (93.00, 121.00)	105.58 (102.81, 108.41)
T_4_ (μg/dL)	222 (100)	95.5	7.38 (6.18, 8.52)	7.26 (7.04, 7.48)
Free T_4_ (ng/dL)	222 (100)	93.2	0.92 (0.82, 1.05)	0.92 (0.90, 0.94)
TBG (μg/mL)	222 (100)	70.7	21.80 (19.23, 24.68)	21.26 (20.66, 21.87)
Lipid metabolism ^d^				
TC (mg/dL)	222 (100)	60.8	190.00 (168.00, 221.00)	190.62 (185.19, 196.21)
HDL-C (mg/dL)	222 (100)	91.0	57.90 (46.78, 69.85)	57.30 (55.27, 59.41)
LDL-C (mg/dL)	222 (100)	70.3	112.00 (90.50, 139.00)	109.16 (104.56, 113.97)
TG (mg/dL)	222 (100)	77.9	100.50 (72.00, 135.75)	106.84 (98.60, 115.77)
CRI-I	222 (100)	82.9	3.32 (2.76, 3.92)	3.33 (3.20, 3.46)
CRI-II	222 (100)	80.6	1.96 (1.46, 2.59)	1.91 (1.81, 2.01)
NHC	222 (100)	48.6	131.30 (107.20, 162.35)	129.88 (124.87, 135.09)
AC	222 (100)	77.0	2.32 (1.76, 2.92)	2.27 (2.14, 2.40)

ND was calculated as half of detection limit. The limit of detection (LOD) for MMP, MEP, MiBP, MnBP, MBzP, MEHP, MEHHP, MEOHP, MECPP, MCMHP, and MiNP were 0.3, 0.3, 1.0, 1.0, 0.3, 0.7, 0.3, 0.3, 0.3, 0.1, and 0.1 ng/mL, respectively

*Abbreviations: GM* Geometric mean, *LOD* Limit of detection, *ND* Not detectable, *MMP* Mono-methyl phthalate, *MEP* Mono-ethyl phthalate, *MiBP* Mono-iso-butyl phthalate, *MnBP* Mono-n-butyl phthalate, *MBzP* Mono-benzyl phthalate, *MEHP* Mono-ethylhexyl phthalate, *MEHHP* Mono-(2-ethyl-5-hydroxyhexyl) phthalate, *MEOHP* Mono-(2-ethyl-5-oxo-hexyl) phthalate, *MECPP* Mono-(2-ethyl-5-carboxypentyl) phthalate, *MCMHP* Mono-(2-carboxymethylhexyl) phthalate, *MiNP* Mono-iso-nonyl phthalate, *TSH* Thyroid-stimulating hormone, *T*_*3*_ Triiodothyronine, *T*_*4*_ Thyroxine, *free T*_*4*_ Free thyroxine, *TBG* Thyroxine-binding globulin, *TC* Total cholesterol, *HDL-C* High-density lipoprotein cholesterol, *LDL-C* Low-density lipoprotein cholesterol, *TG* Triglyceride, *CRI-I* Castelli risk indexes I, *CRI- II* Castelli risk indexes II, *NHC* Non-HDL cholesterol, *AC* Atherogenic coefficient

^a^ The reference ranges (P_95_) of adults for MMP, MEP, MiBP, MnBP, MBzP, MEHP, MEHHP, MEOHP, MECPP, MCMHP, MiNP were 208.2 ng/mL, 265.8 ng/mL, 70.0 ng/mL, 204.3 ng/mL, 11.7 ng/mL, 59.2 ng/mL, 69.7 ng/mL, 35.1 ng/mL, 93.8 ng/mL, 27.7 ng/mL, 12.1 ng/mL. (Liao et al., 2021)

^b^ ΣDEHPm = sum molar concentrations of MEHP + MEHHP + MEOHP + MECPP + MCMHP; ΣDBPm = sum molar concentrations of MiBP + MnBP

^c^ The laboratory reference ranges of adults for TSH, T_3_, T_4_, free T_4_, and TBG were 0.35–4.94 μIU/mL, 58–159 ng/dL, 4.87–11.72 μg/dL, 0.70–1.48 ng/dL, and 15.8–25.4 μg/ mL, respectively

^d^ The laboratory reference ranges of adults for TC, HDL-C, LDL-C, and TG were < 200 mg/dL, > 40 mg/dL, < 130 mg/dL, and < 150 mg/dL, respectively. The reference ranges of adults for CRI-I, CRI-II, NHC, and AC were CRI-I < 4.5 (male), CRI-I < 4.0 (female), CRI-II < 3.0 (male), CRI-II < 2.5 (female), NHC < 130 mg / dL, and AC≦3.0. (Millán et al., 2009; Harari et al., 2017; Bhardwaj et al., 2013)

### Associations of urinary phthalate metabolites levels with thyroid hormones and lipid metabolism indicators

Regarding the associations between urinary lipid metabolism indicators and phthalate metabolites, MMP was negatively associated with LDL-C (β = − 0.036, 95% confidence interval [CI] = − 0.065, − 0.007). MEP exhibited negative association with TC and NHC (TC: β = − 0.022, 95% CI = − 0.041, − 0.004; NHC: β = − 0.025, 95% CI = − 0.050, 0.000), and ΣDEHPm had positive association with HDL-C (β = 0.058, 95% CI = 0.008, 0.109) (Table 3). Regarding associations between thyroid hormones and urinary phthalate metabolites, MnBP was positively associated with T_3_ and free T_4_ (T_3_: β = 0.027, 95% CI = 0.004, 0.050; free T_4_: β = 0.044, 95% CI = 0.019, 0.068), ΣDBPm was negatively associated with T_3_ and free T_4_ (T_3_: β = − 0.061, 95% CI = − 0.103, − 0.019; free T_4_: β = − 0.051, 95% CI = − 0.095, − 0.006), and ΣDEHPm was negatively associated with T_4_ (β = − 0.056, 95% CI = − 0.098, − 0.013) (Table 4).

**Table 3:** Multiple linear regression coefficient (95% CI) for changes in lipid metabolism indicators associated with unit-increases in Ln-phthalate metabolites ^a^

Variables	ln-HDL-C		ln-LDL-C		ln-TC		ln-TG	
	Adjusted β (95%CI)	*P* value	Adjusted β (95%CI)	*P* value	Adjusted β (95%CI)	*P* value	Adjusted β (95%CI)	*P* value
MMP(ng/mL)	−0.011 (− 0.033, 0.010)	0.303	**− 0.036 (− 0.065, − 0.007)**	**0.014** ^*^	− 0.011 (− 0.030, 0.008)	0.267	0.022 (− 0.029, 0.073)	0.390
MEP(ng/mL)	− 0.015 (− 0.036, 0.006)	0.161	− 0.013 (− 0.040, 0.015)	0.368	**−0.022 (− 0.041, − 0.004)**	**0.016** ^*^	−0.032 (− 0.081, 0.017)	0.200
MiBP(ng/mL)	0.007 (−0.017, 0.031)	0.580	−0.005 (− 0.037, 0.026)	0.744	0.007 (− 0.014, 0.028)	0.510	0.005 (− 0.052, 0.061)	0.874
MnBP(ng/mL)	0.006 (− 0.027, 0.039)	0.714	−0.013 (− 0.056, 0.030)	0.550	− 0.012 (− 0.041, 0.017)	0.417	0.017 (− 0.059, 0.093)	0.656
ΣDBPm (n mole/mL) ^b^	− 0.025 (− 0.085, 0.036)	0.421	0.061 (− 0.017, 0.140)	0.125	0.025 (− 0.027, 0.078)	0.344	− 0.034 (− 0.173, 0.106)	0.635
ΣDEHPm (n mole/mL) ^b^	**0.058 (0.008, 0.109)**	**0.024** ^*^	0.005 (− 0.061, 0.071)	0.889	0.039 (− 0.005, 0.084)	0.085	0.063 (− 0.055, 0.180)	0.293
Variables	ln-CRI-I		ln-CRI-II		ln-NHC		ln-AC	
	Adjusted β (95%CI)	*P* value	Adjusted β (95%CI)	*P* value	Adjusted β (95%CI)	*P* value	Adjusted β (95%CI)	*P* value
MMP(ng/mL)	0.001 (−0.023, 0.024)	0.960	−0.024 (− 0.057, 0.008)	0.142	− 0.014 (− 0.040, 0.012)	0.279	−0.002 (− 0.037, 0.031)	0.875
MEP(ng/mL)	−0.008 (− 0.031, 0.015)	0.506	0.002 (− 0.029, 0.034)	0.879	**−0.025 (− 0.050, 0.000)**	**0.048** ^*^	−0.010 (− 0.043, 0.023)	0.549
MiBP(ng/mL)	0.0003 (−0.026, 0.027)	0.981	−0.012 (− 0.048, 0.024)	0.511	0.006 (− 0.023, 0.034)	0.702	−0.001 (− 0.039, 0.036)	0.947
MnBP(ng/mL)	−0.018 (− 0.053, 0.018)	0.319	−0.019 (− 0.068, 0.030)	0.441	−0.019 (− 0.058, 0.019)	0.326	−0.025 (− 0.076, 0.025)	0.327
ΣDBPm (n mole/mL)^b^	0.050 (−0.015, 0.115)	0.132	0.086 (−0.004, 0.175)	0.060	0.052 (−0.019, 0.123)	0.150	0.076 (−0.017, 0.170)	0.108
ΣDEHPm (n mole/mL)^b^	−0.019 (− 0.074, 0.036)	0.489	− 0.054 (− 0.129, 0.022)	0.162	0.023 (− 0.037, 0.082)	0.453	−0.036 (− 0.114, 0.043)	0.372

* < 0.05; ** < 0.01

^a^ Adjusted for age, sex, BMI, cigarette smoking, and urinary creatinine levels

^b^ΣDEHPm = sum molar concentrations of MEHP + MEHHP + MEOHP + MECPP + MCMHP; ΣDBPm = sum molar concentrations of MiBP + MnBP

**Table 4:** Multiple linear regression coefficient (95% CI) for changes in serum thyroid hormones associated with unit-increases in Ln-phthalate metabolites ^a^

Variables	ln-T_3_		ln-T_4_		ln-free T_4_		ln-TSH	
	Adjusted β (95%CI)	*P* value	Adjusted β (95%CI)	*P* value	Adjusted β (95%CI)	*P* value	Adjusted β (95%CI)	*P* value
MMP(ng/mL)	0.006 (−0.010, 0.021)	0.455	0.009 (−0.009, 0.028)	0.329	−0.007 (− 0.024, 0.009)	0.374	−0.012 (− 0.070, 0.045)	0.670
MEP(ng/mL)	0.004 (−0.011, 0.019)	0.576	−0.012 (− 0.029, 0.006)	0.195	0.008 (− 0.008, 0.024)	0.313	0.037 (− 0.019, 0.092)	0.195
MiBP(ng/mL)	0.011 (−0.006, 0.028)	0.195	−0.017 (− 0.037, 0.004)	0.110	0.007 (− 0.011, 0.026)	0.423	0.029 (− 0.035, 0.093)	0.368
MnBP(ng/mL)	**0.027 (0.004, 0.050)**	**0.022** ^*^	−0.026 (− 0.054, 0.001)	0.062	**0.044 (0.019, 0.068)**	**< 0.001** ^**^	0.047 (−0.039, 0.134)	0.280
ΣDBPm (n mole/mL)^b^	**−0.061 (− 0.103, − 0.019)**	**0.005** ^**^	0.041 (− 0.009, 0.092)	0.107	**−0.051 (− 0.095, − 0.006)**	**0.027** ^*^	−0.085 (− 0.243, 0.073)	0.290
ΣDEHPm (n mole/mL)^b^	−0.013 (− 0.048, 0.022)	0.466	**−0.056 (− 0.098, − 0.013)**	**0.011** ^*^	−0.021 (− 0.058, 0.017)	0.282	0.010 (− 0.123, 0.143)	0.880

* < 0.05; ** < 0.01

^a^ Adjusted for age, sex, BMI, cigarette smoking, TBG levels, and urinary creatinine levels

^b^ΣDEHPm = sum molar concentrations of MEHP + MEHHP + MEOHP + MECPP + MCMHP; ΣDBPm = sum molar concentrations of MiBP + MnBP

### Relationship between lipid metabolism indicators and thyroid function

The associations of lipid metabolism indicators with thyroid hormones after adjustment for sex, age, BMI, TBG levels, and smoking are present in Table 5. T_4_ and HDL-C had a significant negative association (β = − 0.284, 95% CI = − 0.440, − 0.128). Positive associations were noted between T_4_ and CRI-I (β = 0.245, 95% CI = 0.074, 0.415), T_4_ and CRI-II (β = 0.265, 95% CI = 0.025, 0.506), and T_4_ and AC (β = 0.370, 95% CI = 0.126, 0.613). Negative associations were observed between free T_4_ and AC (β = − 0.278, 95% CI = − 0.549, − 0.006).

**Table 5:** Multiple linear regression coefficient (95% CI) for changes in lipid metabolism indicators associated with unit-increases in Ln-serum thyroid hormones levels ^a^

Variables	ln-HDL-C		ln-LDL-C		ln-TC		ln-TG	
	Adjusted β 95%CI	*P* value	Adjusted β 95%CI	*P* value	Adjusted β 95%CI	*P* value	Adjusted β 95%CI	*P* value
T_3_	−0.031 (− 0.218, 0.156)	0.743	0.064 (− 0.195, 0.323)	0.627	0.028 (− 0.147, 0.202)	0.754	− 0.062 (− 0.506, 0.383)	0.784
T_4_	**− 0.284 (− 0.440, − 0.128)**	**< 0.001** ^**^	−0.018 (− 0.234, 0.197)	0.866	−0.039 (− 0.184, 0.106)	0.595	0.185 (− 0.185, 0.556)	0.326
Free T_4_	0.094 (−0.080, 0.268)	0.288	−0.038 (− 0.279, 0.202)	0.753	−0.094 (− 0.256, 0.068)	0.256	−0.144 (− 0.557, 0.269)	0.492
TSH	−0.013 (− 0.064, 0.039)	0.634	0.029 (− 0.042, 0.101)	0.422	0.017 (− 0.031, 0.065)	0.481	0.022 (− 0.101, 0.145)	0.725
	ln-CRI-I		ln-CRI-II		ln-NHC		ln-AC	
	Adjusted β 95%CI	*P* value	Adjusted β 95%CI	*P* value	Adjusted β 95%CI	*P* value	Adjusted β 95%CI	*P* value
T_3_	0.059 (−0.145, 0.263)	0.570	0.095 (−0.194, 0.384)	0.516	0.056 (−0.175, 0.287)	0.635	0.087 (−0.205, 0.379)	0.558
T_4_	**0.245 (0.074, 0.415)**	**0.005** ^**^	**0.265 (0.025, 0.506)**	**0.031** ^*^	0.086 (−0.107, 0.278)	0.380	**0.370 (0.126, 0.613)**	**0.003** ^**^
Free T_4_	−0.188 (− 0.377, 0.002)	0.053	− 0.132 (− 0.401, 0.136)	0.332	−0.184 (− 0.398, 0.031)	0.093	**−0.278 (− 0.549, − 0.006)**	**0.045** ^*^
TSH	0.030 (− 0.027, 0.086)	0.299	0.042 (− 0.038, 0.122)	0.303	0.034 (− 0.030, 0.097)	0.301	0.046 (− 0.034, 0.127)	0.261

* < 0.05; ** < 0.01

^a^ Adjusted for age, sex, BMI, cigarette smoking, and TBG

### Mediation analysis and BKMR results

**Fig. 2:**
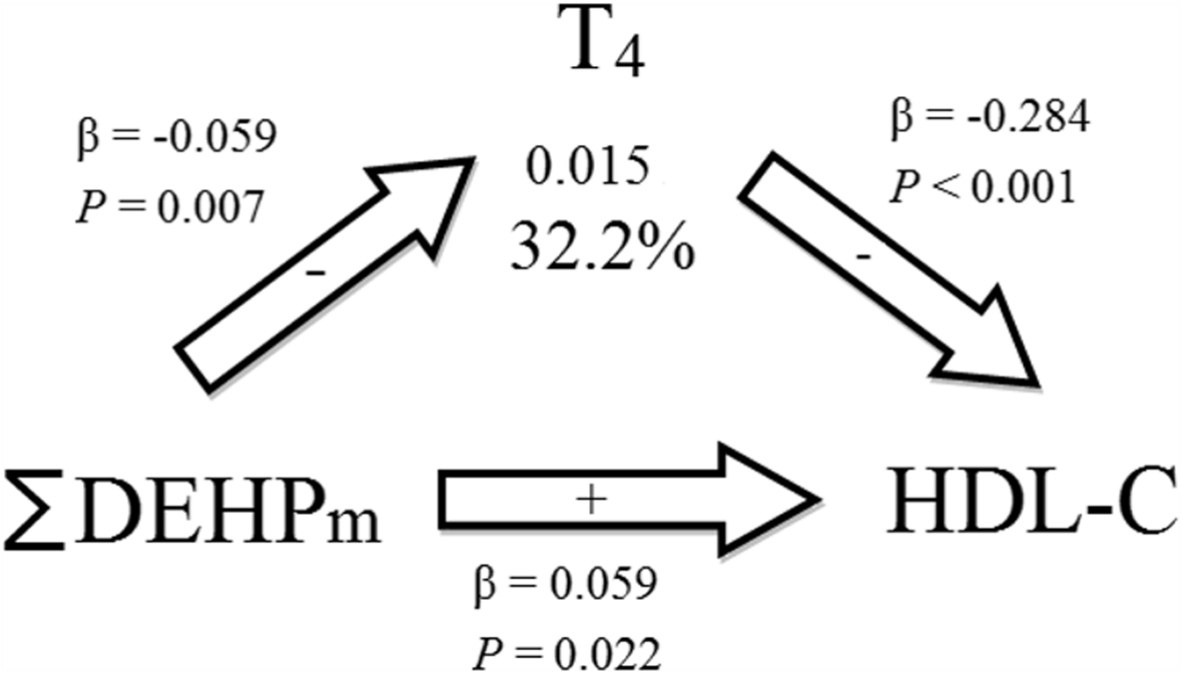
Mediation effects of phthalate exposure on HDL-C through thyroxine (T_4_). The signs in the arrows represent the direction of association

If exposure of phthalate metabolite was significantly associated with thyroid hormones and lipid metabolism indicators that had previously been significantly associated with each other, we then performed mediation analysis. Table 6 presents the results of the mediation analysis. T_4_ mediated 32.2% of the association between ∑DEHPm and HDL-C (indirect effect = 0.015, 95% CI = − 0.0087, 0.05) (Fig. 2). However, the mediation effect was not significant.

**Table 6:** Mediation effects of exposure to phthalates on the homeostatic model assessment of estimated lipid metabolism indicators through thyroid hormones ^a^

Exposure and outcome	Mediator	Estimate indirect effect (95% CI)	Estimate direct effect (95% CI)	Estimated proportion mediated
∑DEHPm and HDL-C	T_4_	0.015 (− 0.0087, 0.05)	0.025 (− 0.0282, 0.08)	32.2%
∑DEHPm and CRI-I	T_4_	−0.019 (− 0.0535, 0.01)	0.001 (− 0.0556, 0.06)	46.8%
∑DEHPm and CRI-II	T_4_	−0.031 (− 0.0790, 0.00)	−0.042 (− 0.1236, 0.04)	38.9%
∑DEHPm and AC	T_4_	−0.031 (− 0.0813, 0.01)	−0.008 (− 0.0910, 0.07)	50.3%
∑DEHPm and CRI-I	Free T_4_	−0.001 (− 0.0150, 0.01)	−0.023 (− 0.0775, 0.03)	3.7%
∑DEHPm and AC	Free T_4_	−0.001 (− 0.0203, 0.02)	−0.038 (− 0.1156, 0.04)	2.2%

* < 0.05; ** < 0.01

^a^ Adjusted for adjusted for age, sex, BMI, cigarette smoking, TBG levels, and urinary creatinine levels

We also used BKMR to explore the associations between lipid metabolism indicators and phthalate exposure, thyroid hormones and phthalate exposure, and lipid metabolism indicators and thyroid function. We observe similar associations with multiple linear regressions (Table S1, S2 and S3, Figs. S1, S2 and S3).

## Discussion

In this study, we observed that the urinary phthalate metabolite ∑DEHPm was negatively associated with HDL-C, ∑DEHPm exhibited a significant negative association with T_4_, and T_4_ showed a significant negative association with HDL-C. These findings suggest that environmental phthalate exposure alters thyroid hormone levels, which in turn impacts lipid metabolism homeostasis.

For associations between urinary phthalate metabolites and lipid metabolism indicators, we observed positive associations between ΣDBPm and LDL-C, ΣDBPm and CRI-I, ΣDBPm and CRI-II, ΣDBPm and NHC, and ΣDEHPm and HDL-C; whereas negative associations were observed between MMP and LDL-C, MEP and TC, and MEP and NHC. However, Olsen et al., in a survey of 70-year-old people in Uppsala, Sweden, observed a positive association between MMP and LDL-C [41]. Perng et al. reported that ΣDBPm was correlated with lower LDL-C in boys enrolled by the Early Life Exposure in Mexico to Environmental Toxicants project from 1997 to 2004 [42]. Yaghjyan et al. found no significant associations of phthalate metabolite concentrations with TC, TG, HDL-C, or LDL-C in a study that enrolled adult women aged ≧18 years and was based on 1999–2004 US National Health and Nutrition Examination Survey data [43]. Dong et al. observed that the concentrations of MCMHP and MEHHP were positively associated with TC and TG, whereas the concentration of MECPP was negatively associated with TC, TG, and LDL-C in > 50-year-old patients with diabetes from Shanghai, China [44]. Variations of the associations between urinary phthalate metabolites and lipid metabolism indicators could be due to differences in study design and participants (e.g. sex, age, patient).

Regarding associations between thyroid hormones and lipid metabolism indicators, we observed that T_4_ had a significant negative association with HDL-C, free T_4_ had significant a negative association with AC, and T_4_ had positive associations with CRI-I, CRI-II, and AC. We did not observe significant associations of free T_4_ with HDL-C, LDL-C, TG, or TC; TSH also was not significantly associated with HDL-C, LDL-C, TG, or TC. Studies have revealed similar results [45–52]. For example, Ren et al. discovered that free T_4_ was not significantly correlated with HDL-C, LDL-C, TG, or TC [49]; Roef et al. showed that free T_4_ was not significantly correlated with HDL-C, LDL-C, or TC, and that TSH was not significantly correlated with LDL-C or HDL-C [50]. However, some studies have revealed different findings [45, 46, 48, 50–58]. For example, Roos et al. observed that free T_4_ had negative associations with TG, TC, HDL-C, and LDL-C [45]; Chin et al. observed that free T_4_ had significant positive associations with TC, HDL-C, and LDL-C [55]; Roef et al. noted positive associations between TSH and TC, TG [50]. Few studies have reported on the associations of T_3_ or T_4_ with TG, TC, HDL-C, and LDL-C. Gutch et al. demonstrated that T_4_ was not significantly correlated with TG, TC, LDL-C, or HDL-C [56]; Roef et al. reported a positive correlation between T_3_ and TG [50]; Kim et al. found that T_3_ was positively correlated with TG and HDL-C [59]; and Roef et al. discovered a positive association between T_4_ and body fat [60]. Variations of the associations between thyroid hormones and lipid metabolism indicators could be due to differences in study design and participants (e.g. age, location, patient).

Animal studies have shown that phthalates such as DEHP and DEP may affect lipid metabolism by binding to the PPAR (e.g., Pradhan et al. [61]). PPAR can be divided into three types: α, δ, and γ. Receptor γ regulates glucose metabolism and fatty acid storage [8], whereas receptor α plays a pivotal role in liver peroxidase proliferation [7, 62]. DEHP causes lipid metabolism disorders by activating the PPARα or PPARγ signal transducer and farnesoid X receptor or liver X receptor signaling pathway, respectively [63]. DBP can activate the PPARα signaling pathway and affect the expression of fatty acid synthase, sterol regulatory element binding proteins, and glycerol-3-phosphate acyltransferase to cause hyperlipidemia and abnormal liver function [64]. Cellular experiments have demonstrated that higher concentrations of MEHP in adipose cells interfere with energy metabolism and may accelerate lipolysis [65]. Animal experiments in rats, rabbits and pigs, in which DEHP was added to their diets, revealed metabolic disturbances in their serum cholesterol concentrations and the inhibition of cholesterol synthesis [66]. Other studies have also reported that the binding of DEHP to the PPAR-α receptor may be involved in cholesterol reduction [67, 68]. Studies have suggested that phthalates reduce LDL-C and TG production by interacting with PPAR-α (ligand-activated transcription), and that they modulate HDL-C concentrations through the role of the PPAR-α in lipid oxidation and fatty acid synthesis [69, 70]. Some studies have indicated that MEHP drives PPAR-α activation more strongly than MBzP [71, 72]. Evidence suggests that MEHP mediates the differentiation of mature adipocytes by binding to PPAR-γ. In particular, PPAR-γ is involved in the regulation of various processes including adipogenesis, adipocyte proliferation, preadipocyte differentiation, fatty acid uptake, and atherosclerotic plaque formation [73]. Exposure to phthalates may affect the regulation of lipid metabolism and even cardiac metabolism by interacting with PPAR, which promotes fat formation and inhibits cholesterol synthesis.

Thyroid hormones regulate various metabolisms in the body. A study suggested that thyroid hormones have a dual regulatory effect on lipid metabolism, stimulating lipid synthesis and promoting lipolysis [16]. Thyroid hormones play important roles in de novo lipogenesis, beta-oxidation (fatty acid oxidation), cholesterol metabolism, and carbohydrate metabolism [74–76]. Thyroid hormones may stimulate the biosynthesis of cholesterol, which is converted to bile acids via the LDL receptor (LDLr) in the liver to regulate serum cholesterol concentrations [77]. TSH may act directly on adipocytes. When TSH binds to receptors in adipocytes, TSH stimulates the release of interleukin-6 (IL-6) from adipocytes, which in turn affects the proliferation of preadipocytes and adipocytes, as well as differentiation and leptin secretion [78]. Thyroid function indicators such as T_3_, T_4_, free T_4_ and TSH may affect the balance of lipid metabolism and energy through a variety of complex biological pathways.

The aforementioned biological mechanisms suggest a direct effect of phthalate exposure on lipid metabolism and the effects of thyroid function indicators on lipid metabolism indicators. However, to date, no animal study or other work has described whether the biological mechanisms underlying the associations between phthalate exposure and lipid metabolism indicators are influenced by thyroid function indicators. In this study, we observed that the thyroid function indicator T_4_ mediates the association between ∑DEHPm and the lipid metabolism indicator HDL-C. The biological mechanisms underlying the effects of phthalate exposure on T_4_ have been described by several studies. For example, some studies have contended that DEHP reduces the expression of sodium-iodine cotransport proteins or that it has an antagonistic effect on thyroid hormones, resulting in decreased T_4_ concentrations [79, 80]. Thyroid hormones also play a role in the synthesis and decomposition of lipids [16]. Furthermore, previous studies indicated that increased levels of LDL-C and HDL-C in the serum can be associated with hypothyroidism, whereas their levels are decreased in hyperthyroidism [81]. These results could support our findings that T_4_ levels were negatively associated with the levels of HDL-C in the serum. CRI-I and CRI-II, having HDL-C as denominator, would exhibit positive associations with T_4_. Taken together, our mediation analysis also supports the assumption that T_4_ could lay the role of mediator in the associations between phthalate exposure and lipid metabolism. Future studies are required to investigate whether a complex pathway is involved in this association.

White adipose tissue (WAT) and brown adipose tissue (BAT) are the two types of adipose tissue found in mammals. BAT inhibits obesity by metabolizing lipids via uncoupling protein 1-mediated uncoupled respiration, whereas WAT accumulates lipids. BAT is involved in the thermogenic response and energy balance regulation in small mammals. Furthermore, BAT activation increases energy expenditure, lowers adiposity, and protects against diet-induced obesity [82]. A recent study found that MEHP and DEHP caused browning-like effects on adipocytes and mice, respectively [83]. Their findings support the browning activity of PAEs both in vivo and in vitro. Browning effects suggest that excess energy in WAT is being dissipated as heat instead of being stored. The browning of WAT is generally thought to help improve metabolic disorders by increasing energy expenditure and decreasing adiposity. MEHP/DEHP could be both endocrine disrupting chemicals and browning chemicals, which seemed contradictory when considered combined [84]. In the present study, ΣDEHPm is positively correlated with HDL-C, which is consistent with the findings in Hsu et al. that DEHP cause browning-like effects on lean mice [83, 84]. Further study on browning-like effects with DEHP or DEHP alternatives (e.g. DBP, etc.) would be warranted.

Our study’s key strength is its novel approach to mediation analysis, with the associations between phthalate exposure, lipid metabolism, and thyroid function, along with possible mediating effects, being investigated. To the best of our knowledge, no other study has investigated the association between phthalate exposure and lipid metabolism indicators and whether this association is mediated by thyroid function indicators. Therefore, in addition to examining the correlation between phthalate exposure and lipid metabolism indicators in Taiwanese adults, this study investigated thyroid function indicators as potential mediators of the relationship between phthalate exposure and lipid metabolism indicators in Taiwanese adults. We collaborated with the NAHSIT team and used the same sampling frame and procedure in subject recruitment in order to obtain sufficient participation from the general Taiwanese population. We followed NAHSIT’s Standard Operating Procedure [85, 86], transported and stored samples at − 80 °C until analysis. We analyzed the sample within weeks or 2–3 months just after the samples were collected, and did quality control in phthalate metabolite analysis. By these systematic methods in subject selection, sample collection, and quality, we were able to reduce variation and increase reliability of final analysis. This study has the following limitations. First, this is the cross-sectional study, which could not explain causality. Second, phthalate exposure measurements were derived from a single urine test and were corrected for the creatinine concentration, but they may not be representative of the participants’ complete exposure to phthalates over time. However, previous studies have indicated that a single urine test is representative of phthalate exposure over a period of 16 weeks to 6 months [87, 88]. Third, the blood samples analyzed in this study were collected only once, and thyroid hormone concentrations may have vary among individual participants over time. Nevertheless, Andersen et al. suggested that the measured values of thyroid hormones in individuals do not fluctuate greatly over time [89]. Third, data on iodine or selenium exposure concentrations were not available for the study population, and deficiencies in these trace elements may have affected the presence of abnormal thyroid function indicators [90]. But, individuals with self-reported endocrine system abnormalities (e.g., abnormal thyroid function or diabetes mellitus) were excluded from this study, and approximately 90% of the study population exhibited thyroid function values that were within the reference range. Finally, though our subjects were chosen from general population, our results were limited for Taiwanese and could not represent the whole general population. Further study with larger sample size would be needed to elucidate the associations.

## Conclusion

Our findings supported that thyroid hormones mediate the association between phthalate exposure and lipid metabolism. In particular, T_4_ levels strongly mediate the effect of phthalate exposure on HDL-C in humans. Large-scale epidemiological and mechanistic research is required to validate these associations and determine the underlying biological mechanisms.

## Supplementary Information

**Additional file 1.**

## Data Availability

The datasets used and/or analysed during the current study are available from the corresponding author on reasonable request.
