# Supplementary material for: Mediation effects of thyroid function in the associations between phthalate exposure and lipid metabolism in adults
Source: Environ Health. 2022 Jul 1;21:61. doi: 10.1186/s12940-022-00873-9 (PMC9248169; doi:10.1186/s12940-022-00873-9)
Supplement: Supplementary file 1 — Additional file 1. [file 12940_2022_873_MOESM1_ESM.docx]

Supplementary Materials

Detection process of phthalate metabolites and thyroid function indicators

Eleven phthalate metabolites including mono-ethylhexyl phthalate (MEHP), mono-(2-ethyl- 5-oxo-hexyl) phthalate (MEOHP), mono-(2-ethyl-5-hydroxyhexyl) phthalate (MEHHP), mono-(2-ethyl-5-carboxypentyl) phthalate (MECPP), mono-(2-carboxymethylhexyl) phthalate (MCMHP), mono-n-butyl phthalate (MnBP), mono-iso-butyl phthalate (MiBP), monoethyl phthalate (MEP), mono-iso-nonyl phthalate (MiNP), mono-benzyl phthalate (MBzP) and mono-methyl phthalate (MMP) representing exposure to seven commonly used phthalates (DEHP, DnBP, DiBP, DEP, di-iso-nonyl phthalate (DiNP), benzyl butyl phthalates (BBzP) and dimethyl phthalate (DMP) were measured in each urine sample. We used a modified method previously described by Koch et al. (2003) using on-line chromatography to increase the efficiency and accuracy of analysis. After a urine sample was thawed and sonicated for 10–15 min, the urine sample (100 μl) was loaded into a glass vial (2 ml) which contained ammonium acetate (AA, 20 μl, >98%, Sigma Aldrich Lab., Inc., St. Louis, MO, USA), β-glucuronidase (10 μl, E.coli K12, Roche Biomedical, Mannheim, Germany), and a mixture of ten isotopic (13C4) phthalate metabolite standards (100 μl. Cambridge Isotope Lab., Inc., Andover, MA, USA). After the sample was incubated (37℃, 90 min), a 270 μl solution (5% ACN), Merck, Darmstadt, Germany) with 0.1% formic acid (FA, Merck, Darmstadt, Germany) was added and sealed with the PTEF cap for analysis. We used an on-line system which was coupled with liquid chromatography/electrospray tandem mass spectrometry (LC–ESI-MS/MS) (Agilent 1200/ API 4000, Applied Biosystems, Foster City, CA, USA). We used two columns in our online system. One C18 column (Inertsil ODS-3, 33×4.6 mm, 5 μm, GL Science, Tokyo, Japan) was used to extract and clean our sample, and an analytical column (Inertsil Ph, 150 *4.6 mm, 5 μm, GL Science, Tokyo, Japan) was used to separate different phthalate metabolites. The gradient program of the clean-up column was listed as follows: 100% solution A (5% ACN + 0.1% FA) (0–7 min), 100% solution B (90% ACN + 0.1% FA) (7–9 min), 100% solution A (9–10 min) and continued to 12 min. The flow rate was set at 1000 μl/min. The analytical column gradient program was listed as follows: 100% solution C (50% ACN + 10 mmole AA) (0–3.6 min), 100% solution D (95% ACN + 10 mmole AA) (3.6–8.6 min), 100% solution C (8.6–9 min) and continued to 12 min. We used a negative multiple reaction monitoring model for mass spectroscopy detection. The ion pair of each phthalate metabolites was listed as follows: MEHP (277/134), MEOHP (291/143), MEHHP (293/121), MECPP (307/159), MCMHP (307/ 113), MnBP (221/71), MiBP (221/71), MEP (193/121), MiNP (291/121), MBzP (255/183), and MMP (179/107). The detection limits of MEHP, MEOHP, MEHHP, MECPP, MCMHP, MnBP, MiBP, MEP, MiNP, MBzP, and MMP were 0.7, 0.3, 0.3, 0.3, 0.1, 1, 1, 0.3, 0.1, 0.3 and 0.3 ng/mL respectively. One blank, repeat, and quality control (QC) sample were included in each batch of analyzed samples. The concentration of blank samples shall be below two times the detection limit. The QC sample was spiked in a pooled urine sample with a mixture of phthalate metabolite standards (20–50 ng/ml) in each sample. The relative percent difference of repeat sample and recovery of QC sample shall be within ± 30%. Urinary creatinine levels were measured by spectrophotometric methods, with picric acid as a reactive compound for reading at 520 nm measurement (Beckman; DXC 800).

Thyroid function was measured as the serum concentrations of thyroxine (T4), free T4, triiodothyronine (T3), thyroid- stimulating hormone (TSH), and thyroxine-binding globulin (TBG). The morning blood sample from each participant was collected and immediately centrifuged for 20 min at 4 °C, and then stored at −80 °C until analysis. The serum levels of T4, T3, free T4, and TSH were quantified using a chemiluminescent microparticle immunoassay on Beckman Coulter's UniCel (R) DxI 800 Immunoassay System (Beckman Coulter Inc., Brea, CA, USA). The assay sensitivities for T3, T4, free T4, and TSH were 0.25 ng/mL, 1.0 μg/dL, 0.4 ng/dL, and 0.0025 μIU/mL, respectively. Serum TBG was measured using the immunoenzymometric assay with ACCUBIND ELISA Microwells (Monobind Inc., Product Code 3525-300). The assay sensitivities for TBG was 1.0 μg/mL.

We add detection process of phthalate metabolites and thyroid function indicators in supplementary materials.

Reference

Koch HM, Gonzalez-Reche LM, Angerer J. On-line clean-up by multidimensional liquid chromatography- electrospray ionization tandem mass spectrometry for high throughput quantification of primary and secondary phthalate metabolites in human urine. J Chromatogr B Analyt Technol Biomed Life Sci. 2003; 784: 169–82.

Table S1. Posterior inclusion probability (PIP) for lipid metabolism indicators associated with Ln-phthalate metabolites. (BKMR)

|  |  |  |  |  |
| --- | --- | --- | --- | --- |
| Variable | ln-HDL-C | ln-LDL-C | ln-TC | ln-TG |
| MMP | 0.000 | 0.013 | 0.000 | 0.054 |
| MEP | 0.001 | 0.001 | 0.000 | 0.017 |
| MiBP | 0.000 | 0.002 | 0.002 | 0.004 |
| MnBP | 0.000 | 0.000 | 0.000 | 0.004 |
| ΣDBPm | 0.000 | 0.001 | 0.001 | 0.009 |
| ΣDEHPm | 0.007 | 0.006 | 0.004 | 0.049 |

|  |  |  |  |  |
| --- | --- | --- | --- | --- |
| Variable | ln-CRI-I | ln-CRI-II | ln-NHC | ln-AC |
| MMP | 0.135 | 0.118 | 0.000 | 0.291 |
| MEP | 0.393 | 0.176 | 0.003 | 0.536 |
| MiBP | 0.163 | 0.071 | 0.012 | 0.177 |
| MnBP | 0.085 | 0.137 | 0.000 | 0.240 |
| ΣDBPm | 0.233 | 0.283 | 0.003 | 0.381 |
| ΣDEHPm | 0.176 | 0.182 | 0.007 | 0.241 |

Table S2. Posterior inclusion probability (PIP) for serum thyroid hormones associated with Ln-phthalate metabolites. (BKMR)

|  |  |  |  |  |
| --- | --- | --- | --- | --- |
| Variable | ln-T_3_ | ln-T_4_ | ln-free T_4_ | ln-TSH |
| MMP | 0.000 | 0.002 | 0.917 | 0.061 |
| MEP | 0.000 | 0.004 | 0.721 | 0.107 |
| MiBP | 0.000 | 0.003 | 0.699 | 0.075 |
| MnBP | 0.000 | 0.015 | 0.978 | 0.088 |
| ΣDBPm | 0.000 | 0.015 | 0.825 | 0.119 |
| ΣDEHPm | 0.006 | 0.702 | 0.843 | 0.230 |

Table S3. Posterior inclusion probability (PIP) for lipid metabolism indicators associated with Ln-serum thyroid hormones. (BKMR)

|  |  |  |  |  |
| --- | --- | --- | --- | --- |
| Variable | ln-HDL-C | ln-LDL-C | ln-TC | ln-TG |
| T_3_ | 0.232 | 0.222 | 0.192 | 0.276 |
| T_4_ | 0.996 | 0.248 | 0.287 | 0.282 |
| Free T_4_ | 0.278 | 0.242 | 0.264 | 0.303 |
| TSH | 0.012 | 0.092 | 0.037 | 0.078 |

|  |  |  |  |  |
| --- | --- | --- | --- | --- |
| Variable | ln-CRI-I | ln-CRI-II | ln-NHC | ln-AC |
| T_3_ | 0.484 | 0.491 | 0.241 | 0.538 |
| T_4_ | 0.866 | 0.708 | 0.263 | 0.897 |
| Free T_4_ | 0.723 | 0.517 | 0.493 | 0.732 |
| TSH | 0.366 | 0.391 | 0.102 | 0.421 |


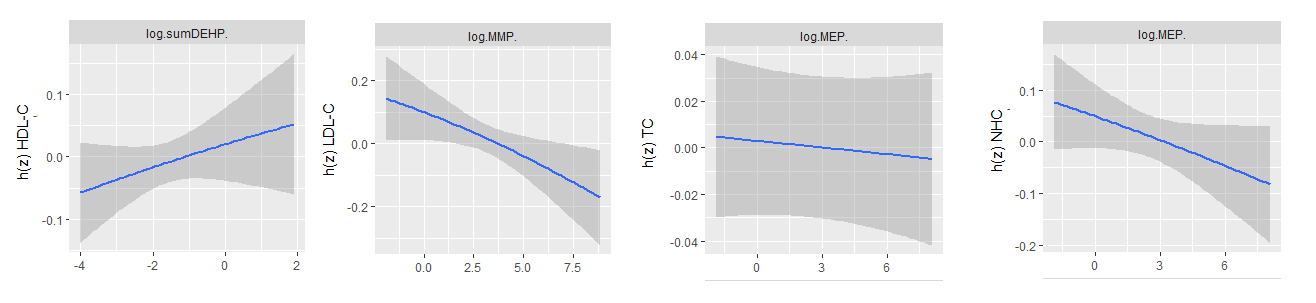


Figure S1. Univariate relationship between Ln-phthalate metabolites and Ln-lipid metabolism indicators. (BKMR)


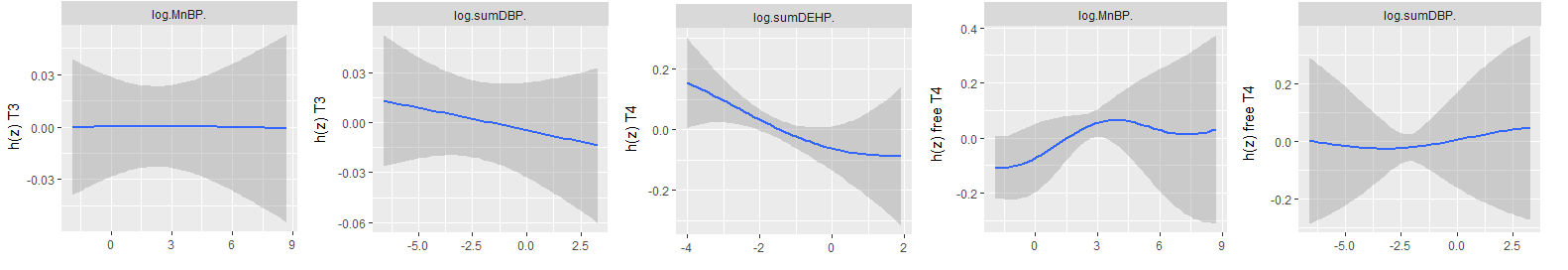


Figure S2. Univariate relationship between Ln-phthalate metabolites and Ln-thyroid hormones. (BKMR)


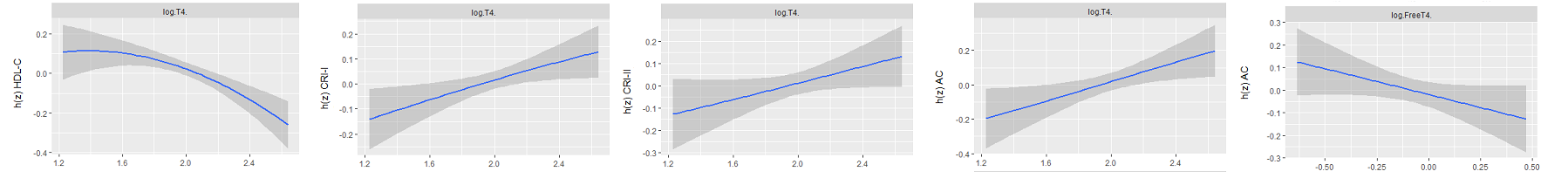


Figure S3. Univariate relationship between Ln-thyroid hormones and Ln-lipid metabolism indicators. (BKMR)
